# Evaluating the GUIDE Model

**DOI:** 10.1002/alz.095814

**Published:** 2025-01-09

**Authors:** Lori Timmins

**Affiliations:** ^1^ Mathematica, Chicago, IL USA

## Abstract

N/A N/A N/A N/A N/A N/A N/A N/A N/A N/A N/A N/A

